# Roles of human POLD1 and POLD3 in genome stability

**DOI:** 10.1038/srep38873

**Published:** 2016-12-15

**Authors:** Emanuela Tumini, Sonia Barroso, Carmen Pérez -Calero, Andrés Aguilera

**Affiliations:** 1 Centro Andaluz de Biología Molecular y Medicina Regenerativa CABIMER, Universidad de Sevilla, Avd. Américo Vespucio s/n, 41092 Seville, Spain

## Abstract

DNA replication is essential for cellular proliferation. If improperly controlled it can constitute a major source of genome instability, frequently associated with cancer and aging. POLD1 is the catalytic subunit and POLD3 is an accessory subunit of the replicative Pol δ polymerase, which also functions in DNA repair, as well as the translesion synthesis polymerase Pol ζ, whose catalytic subunit is REV3L. In cells depleted of POLD1 or POLD3 we found a differential but general increase in genome instability as manifested by DNA breaks, S-phase progression impairment and chromosome abnormalities. Importantly, we showed that both proteins are needed to maintain the proper amount of active replication origins and that POLD3-depletion causes anaphase bridges accumulation. In addition, POLD3-associated DNA damage showed to be dependent on RNA-DNA hybrids pointing toward an additional and specific role of this subunit in genome stability. Interestingly, a similar increase in RNA-DNA hybrids-dependent genome instability was observed in REV3L-depleted cells. Our findings demonstrate a key role of POLD1 and POLD3 in genome stability and S-phase progression revealing RNA-DNA hybrids-dependent effects for POLD3 that might be partly due to its Pol ζ interaction.

DNA replication is an essential process in which DNA is duplicated and passed on to daughter cells, allowing the transmission of genetic information. To safeguard its integrity, cells have developed sophisticated mechanisms that constitute the DNA damage response (DDR) pathway. DNA replication and repair are often tightly interconnected, as first manifested by the dual roles that DNA polymerases (Pol) have on both processes. Pol δ is a clear example of such a dualism^1^. It is a heterotetrameric complex composed of the catalytic subunit POLD1 (p125) and three accessory subunits: POLD2 (p50), POLD3 (p66) and POLD4 (p12). During DNA replication, Pol δ is believed to be responsible for lagging strand DNA synthesis^2^,^3^. In addition, Pol δ has a role in DNA double-strand break (DSB) repair via homologous recombination (HR), in DNA repair synthesis as the major gap-filling polymerase, and in common fragile site instability^4^,^5^. Recent publications have shown the importance of human Pol δ in the DNA damage response (DDR)^6^,^7^,^8^,^9^,^10^. However the regulation and dynamics of these events are still largely unknown in human cells.

POLD3 interacts with PCNA and its affinity for it increases in a phosphorylation-dependent manner^11^. Moreover, in *Saccharomyces cerevisiae* the error-prone translesion synthesis (TLS) mediated by Pol ζ depends on Pol32, the yeast homolog of POLD3^12^,^13^, which has recently been shown to be a key subunit of Pol ζ together with POLD2 in yeast and human cells^14^,^15^,^16^,^17^.

Pol ζ consists of the catalytic subunit REV3L and the accessory subunit REV7 and it is the only TLS polymerase belonging to the B-family of DNA polymerases to which the main replicative polymerases such as Pol δ belong^18^. In addition to its ability to bypass DNA lesions, Pol ζ plays an important role in several DDR pathways such as HR repair, non-homologous end-joining (NHEJ) and interstrand crosslink (ICL) repair^19^. Deletion of REV3L causes embryonic lethality in mice and this subunit seems to own additional functions independent of the accessory subunit REV7, having been reported to be specifically required to prevent common fragile sites expression^20^.

In the budding yeast *Saccharomyces cerevisiae*, deletion of *POL32* is viable, whereas in the fission yeast *Schizosaccharomyces pombe* the homolog *CDC27* is essential^21^. *POL32* deletion causes hyper-sensitivity to DNA damage and synthetic lethality with mutations in genes of the DDR network, suggesting a specific role of Pol32 in repair^12^,^13^. Indeed, Pol32 is required for Break Induced Replication (BIR), the HR pathway repairing one-ended DSBs^22^. A similar role has been proposed recently for human POLD3, whose depletion results in a high frequency of genome duplications^23^. BIR is a relevant physiological process as it could account for chromosomal translocation, extensive loss of heterozygosity or telomere elongation in the absence of telomerase, which are common features of cancer cells^24^,^25^. Recently, a new role has been demonstrated for POLD3 in mitosis, during which it drives DNA repair synthesis following replicative stress^26^.

It has been shown by recent studies that germline mutations or common variations in POLD1 and POLD3 genes predispose to colorectal cancer and other malignancies^27^,^28^,^29^,^30^. Therefore it is of growing importance to expand the knowledge about Pol δ and to further dissect the molecular contribution of its subunits to genome instability.

To better understand the role of human Pol δ in the control of genome stability we evaluated the impact of depleting POLD1 and POLD3 on DNA replication and DDR. We found a general increase in genome instability as determined by DNA breaks accumulation, activation of the DNA damage checkpoint, impaired S-phase progression under replication stress and accumulation of chromosome abnormalities in POLD1- and POLD3-depleted cells. Such deficiencies were accompanied by a decrease in the density of active replication origins, which suggests a key role of POLD1 and POLD3 in this process. Moreover, we observed a variety of common and different phenotypes caused by down-regulation of either the catalytic or the accessory subunits of Pol δ. Interestingly, POLD3 depletion seemed to favor particularly the formation of anaphase bridges that are less prone to form in POLD1-depleted cells, pointing toward a differential function of this subunit in chromosome stability. Furthermore γH2AX foci were rescued by RNase H1 overexpression in POLD3 but not in POLD1-depleted cells, suggesting that POLD3 has a key role in preventing RNA-DNA hybrids-associated genome instability. This latter phenotype could be partly dependent on the Pol ζ-associated role of POLD3 since the increase of γH2AX foci caused by REV3L depletion was recovered by RNase H1 overexpression.

## Results

### Differential impact of POLD1 and POLD3 on DNA replication

**Figure 1:**
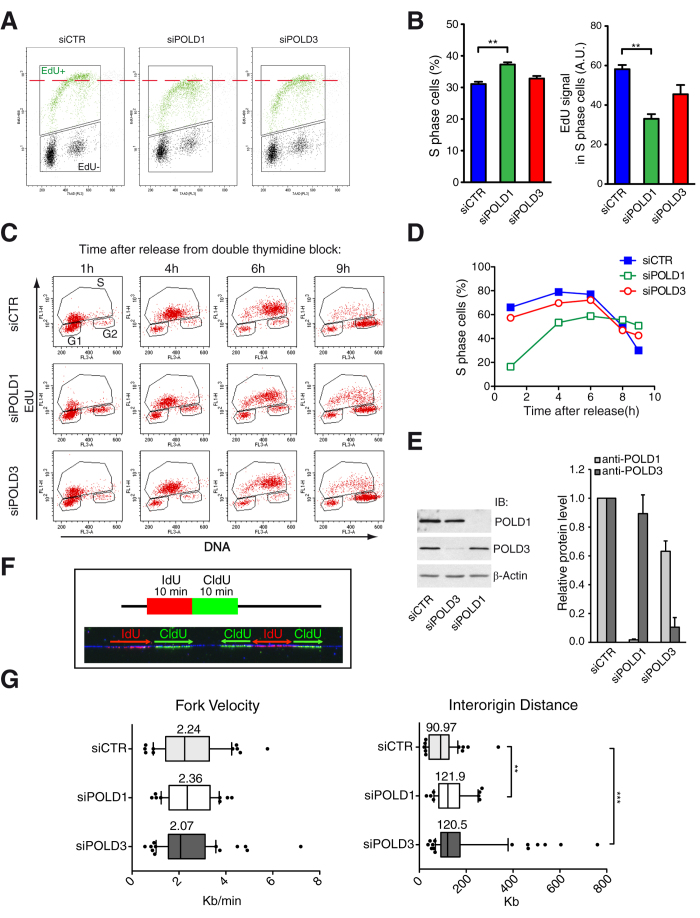
Effect of POLD1 and POLD3 depletion on DNA replication and S-phase progression. (**A**) Representative cell cycle profiles of exponentially growing HeLa cells after the indicated siRNA-mediated depletions. (**B**) Percentage of S-phase cells and quantification of EdU average signal in S-phase cell. More than 1000 cells were scored for each experiment. Means ± SEM from five independent experiments are shown. Differences between distributions were assessed by the Mann-Whitney test. (**C**) FACS profile of HeLa cells at different time points after release from double thymidine block. (**D**) Percentage of cells that progress into S-phase based on panel C. A representative experiment is shown and quantified. (**E**) Immunoblot to assay the knockdown of the siRNA targeted proteins and relative protein quantification. β-Actin was used as a loading control. Densitometric quantification of the corresponding bands was performed using ImageJ analysis software. Values were normalized to β-Actin and expressed as relative to siCTR. Means ± SEM from three independent experiments are shown. (**F**) Diagram and representative picture of a DNA fiber labeled by IdU and CldU for single DNA molecule analysis on HeLa cells. (**G**) Distribution of RF velocity and interorigin distance. The data from two independent experiments were pulled together. Differences between distributions were assessed by the Mann-Whitney test.

To determine the cellular roles of POLD1 and POLD3, we first assayed the effects of their depletion on cell proliferation and S-phase progression. Analysis of cell cycle distribution revealed that POLD1-depleted cells accumulated in S-phase and had a reduced incorporation of the thymidine analogue EdU (Fig. 1A and B), consistent with a diminished performance in DNA replication. Compared to POLD1, POLD3 depletion resulted in a milder decrease of incorporated EdU.

To explore the role of POLD1 and POLD3 in S-phase progression, HeLa cells were synchronized at G1/S phase by a double thymidine block, and after release DNA content was monitored by FACS at different time points (Fig. 1C,D and Supplementary Fig. S1A). While POLD1 depletion greatly affected S-phase progression, POLD3 depletion produced a milder effect that became more severe at the last time points, as if cells took longer to complete the S-phase. To better detect these differences, S-phase cells were gated in three different regions corresponding to early (S1), middle (S2) and late (S3) S-phase accordingly to the DNA content (Supplementary Fig. S1B–D). In this way, we could clearly observe that POLD1 and POLD3-depleted cells took longer to reach the late S-phase (S3).

Next we performed single-molecule analysis of DNA replication by molecular combing (Fig. 1F and G). Neither POLD1 nor POLD3 depletion affected the speed of replication forks (RFs). However, a clear increase in interorigin distance was observed, which may indicate a reduction in the overall firing of replication origins, possibly as a consequence of lower availability of the Pol δ subunits and therefore a lower ability to assemble at new RFs.

We also assessed cell viability within 5 days of POLD1 or POLD3 depletion using the WST-1 colorimetric assay in HeLa cells (Supplementary Fig. S1E). While POLD1 depletion clearly slowed down the proliferation rate, POLD3 only showed a minor defect, suggesting that the role of this subunit may not be strictly necessary for DNA synthesis. Nevertheless, the defect in proliferation caused by POLD1 depletion was not drastic despite the knockdown being efficient (Fig. 1E), and given the fact that in yeast deletion of the *POLD1* homolog *pol3* is lethal^31^. We observed that *POLD3* deletion caused a partial reduction of POLD1 protein level, while POLD3 levels remained virtually unchanged in POLD1-depleted cells (Fig. 1E).

### High DNA damage after POLD1 or POLD3 depletion

**Figure 2:**
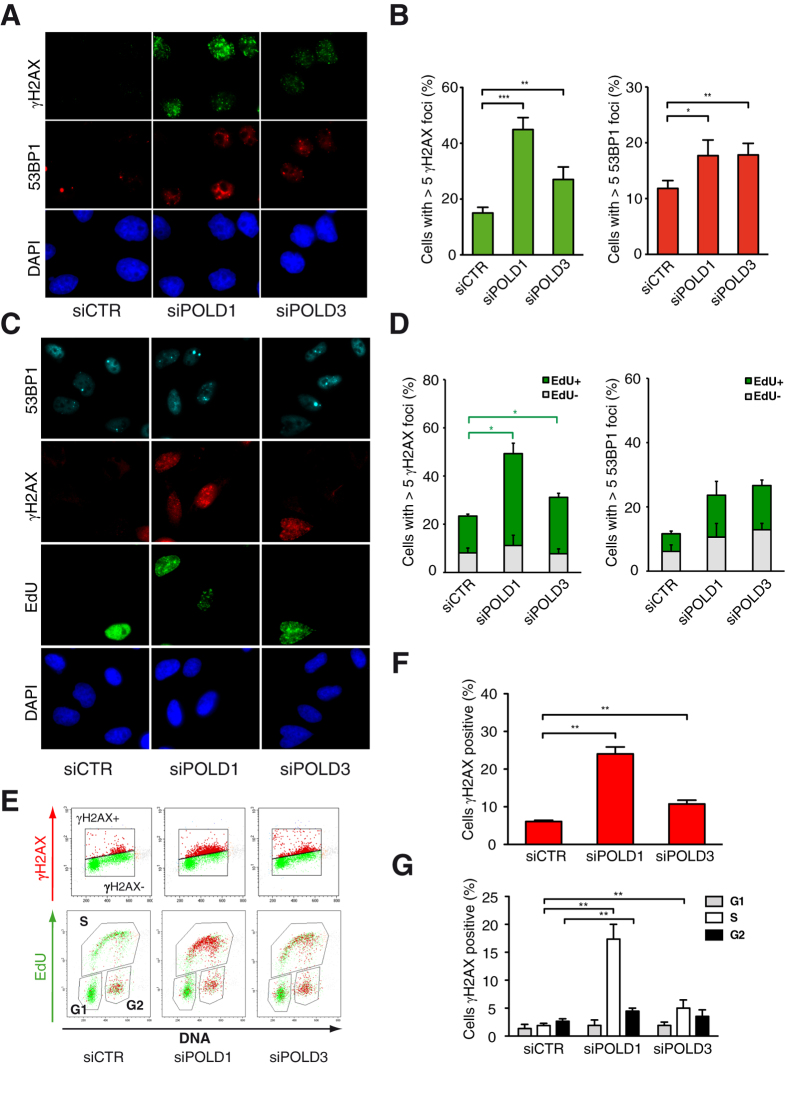
DNA damage markers and their cell cycle distribution in POLD1- and POLD3-depleted cells. (**A**) Immunofluorescence of HeLa cells stained with antibodies against γH2AX and 53BP1. (**B**) Percentage of cells with more than 5 foci. Means ± SEM from at least five independent experiments are shown. (**C**) Immunofluorescence of HeLa cells stained with antibodies against γH2AX and 53BP1 and with EdU click-it labeling kit. (**D**) Percentage of cells with more than 5 foci in EdU positive and EdU negative cells. More than 100 cells were scored for each experiment. Means ± SEM from at least three independent experiments are shown. (**E**) FACS profile from one representative experiment of HeLa cells labeled for γH2AX, EdU and DNA. (**F**) Percentage of γH2AX positive cells. More than 1000 cells were scored for each experiment. Means ± SEM from five independent experiments are shown. (**G**) Percentage of γH2AX positive cells in the different cell cycle phases. More than 1000 cells were scored for each experiment. Means ± SEM from five independent experiments are shown. Differences between distributions were assessed by paired t-test (B) or by the Mann-Whitney test (**D**, **F** and **G**).

Next we analyzed the effect of POLD1 and POLD3 depletion in DDR. In both cases we found an accumulation of foci of H2AX phosphorylated at Ser139 (γH2AX) and of 53BP1, as markers of DSBs that significantly increased with respect to mock-treated cells (Fig. 2A and B). We next assayed whether such DNA breaks were specifically associated with the S-phase. For this purpose cells were labeled with EdU to identify those undergoing DNA replication. The numbers of γH2AX and 53BP1 foci were scored either in EdU-positive or EdU-negative cells (Fig. 2C). The majority of γH2AX foci were observed in S-phase cells, while 53BP1 foci were equally distributed between S and non-S-phase cells (Fig. 2D). In addition, flow cytometry was used to analyze cells simultaneously labeled for DNA content, EdU incorporation and γH2AX (Fig. 2E). With this approach, we also found an increase in γH2AX-positive cells (Fig. 2F) that accumulated mainly in S-phase after both POLD1 and POLD3 depletion, as well as in G2 after POLD1 depletion (Fig. 2G).

### Enhanced SCE and chromosomal abnormalities

**Figure 3:**
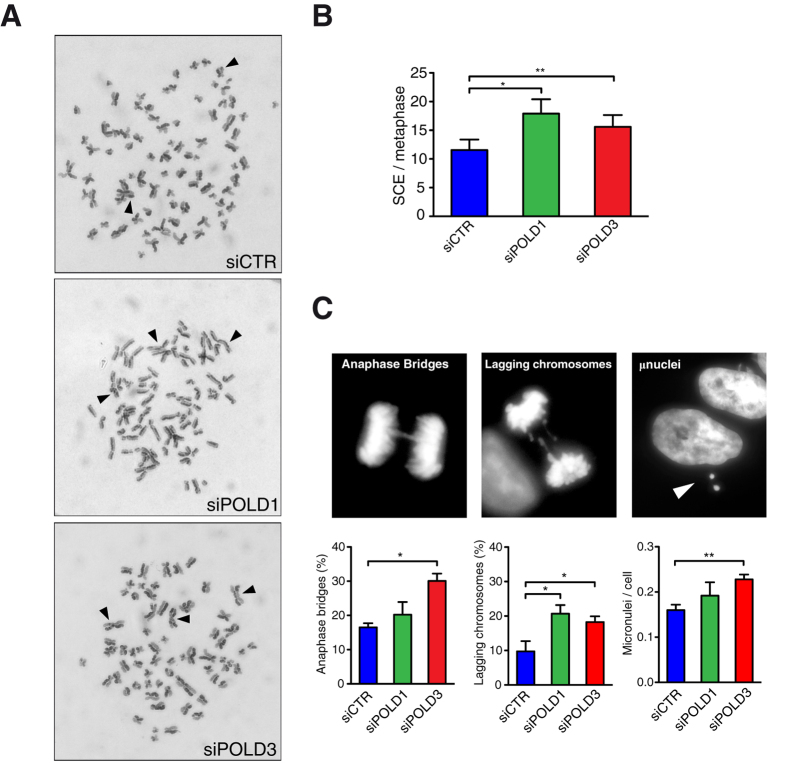
Effect of POLD1 and POLD3 depletion on SCE and chromosome abnormalities. (**A**) Representative images of metaphase spreads with differentially stained chromatids. SCE events are pointed by arrowheads. **(B)** Frequency of SCE per metaphase. More than 20 metaphases were scored for each experiment. Means ± SEM from three independent experiments are shown. Differences between distributions were assessed by paired t-test. **(C)** Representative images of anaphase bridges, lagging chromosomes and micronuclei and their quantification. More than 50 anaphases or 100 cells (for micronuclei) were scored for each experiment. Means ± SEM from at least three independent experiments are shown. Differences between distributions were assessed by the one-tailed Mann-Whitney test.

In addition to DNA breaks, an increase in genome instability can be manifested by different genetic products and chromosomal abnormalities such as sister chromatid exchange (SCE), anaphase bridges and lagging chromosomes^32^. To evaluate the type of instability generated by POLD1 and POLD3 depletion we extended our analysis to this type of hallmark products and found that their depletion caused an increase in SCEs (Fig. 3A and B). This phenotype could be the direct consequence of DNA breaks or gaps, whose repair through HR could generate exchange of genetic material between sister chromatids. We detected higher levels of spontaneous SCE after *POLD3* knockdown also in U2OS cells (Supplementary Fig. S2).

As it can be seen in Fig. 3C, depletion of POLD1 and POLD3 was also associated with a higher incidence of anaphase bridges, lagging chromosomes and micronuclei. Lagging chromosomes can result from failures during mitotic segregation, such as merotelic kinetochore attachment. Alternatively they could derive from breakage of anaphase bridges or could represent chromosome fragments generated by DSBs^33^. Anaphase bridges could arise from dicentric chromosomes that might originate as a consequence of telomere erosion, DSBs or replicative stress. Micronuclei could result as a consequence of both anaphase bridges and lagging chromosomes. Interestingly, POLD3 depletion showed a stronger phenotype of anaphase bridges formation with respect to POLD1 depletion, whose effect was instead quite mild. Conversely, POLD1 depletion increased the frequency of lagging chromosomes more markedly than POLD3. These differences in chromosome abnormalities after POLD3 or POLD1 depletion are of particular interest because they could suggest a specific role of POLD3 over POLD1.

In addition, we could rule out that POLD3 is necessary for the main mechanisms of HR-mediated repair that result in gene conversion. For this purpose we measured HR in the established MCF7 human cell line containing the integrated copy of the pDRGFP reporter (MCF7 DR-GFP)^34^. Following overexpression of the I-*Sce*I endonuclease, the reconstitution of wild-type GFP from two nonfunctional copies of the gene was assessed. POLD3 depletion did not affect the percentage of GFP-positive cells, while POLD1 depletion strongly affected it (Supplementary Fig. S3). This experiment suggests that while POLD1 depletion impaired gene conversion, POLD3 depletion did not.

### Impaired progression through perturbed S-phase

**Figure 4:**
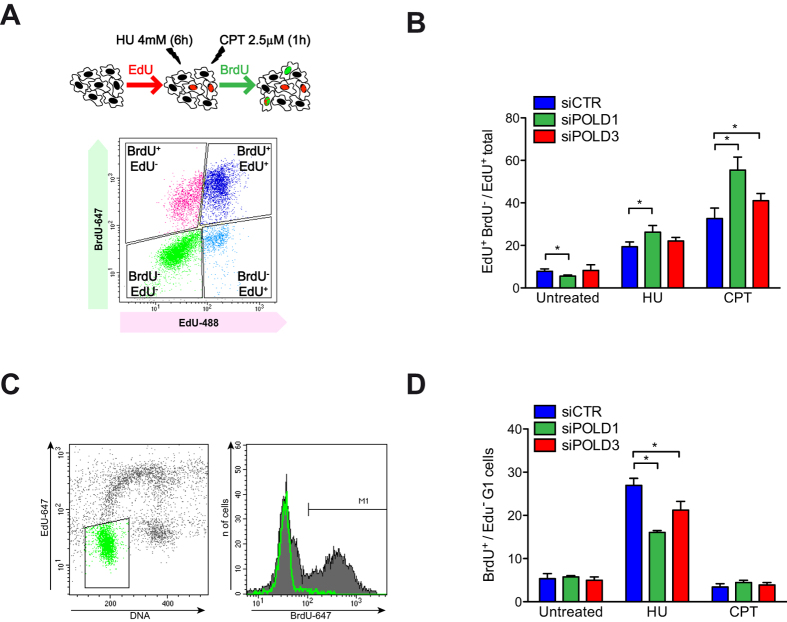
Cytometer-based assay of progression through S-phase after genotoxic stress in POLD1- and POLD3-depleted samples. (**A** and **C**) Outline of the experimental procedure and representative images of the cell cycle profile obtained by FACS. (**B**) Quantification of cells that stop DNA synthesis, based on FACS profiles alike the one shown in panel A. (**D**) Quantification of cells that begin or restore DNA synthesis based on FACS profiles alike the one shown in panel C. More than 1000 cells were scored for each experiment. Means ± SEM from four independent experiments are shown. Differences between distributions were assessed by the Mann-Whitney test.

We analyzed next the impact of POLD1 and POLD3-depletion on S-phase progression under replication stress conditions. For this purpose we used a cytometer-based assay: cells were first pulse-labeled with EdU, treated with hydroxyurea (HU) or camptothecin (CPT) and released in the presence of BrdU (Fig. 4A). In this assay, cells incorporating the first nucleotide but not the second (EdU^+^ BrdU^−^) were considered to have stopped replication, either as a consequence of a replication block or because they had completed the S-phase. During unperturbed growth conditions, the EdU^+^ BrdU^−^ cells could be considered the group of cells completing replication. The fraction of EdU^+^ BrdU^−^ cells decreased after POLD1-depletion, indicating that fewer cells had completed replication most likely as a consequence of a slower progression through S-phase (Fig. 4B). Following treatment with HU or CPT, the EdU^+^ BrdU^−^ cell population would be mainly constituted by cells unable to carry out replication. As expected, this cell population increased in the siCTR, and to a higher extent in POLD1- and POLD3-depleted cells, showing the stronger and statistically significant effect when cells were treated with CPT (Fig. 4B).

We then evaluated the ability of G1 cells to enter the S-phase after replication block or genotoxic treatment. Using the same flow cytometry-based assay, we measured the amount of cells that were able to incorporate the second nucleotide analogue (BrdU^+^) considering the cells in G1 at the time of treatment (EdU^−^, 2c DNA content) (Fig. 4C and D). After HU treatment, cells would accumulate at the beginning of the S-phase, right after origins have already been fired, so that BrdU^+^ cells would be the cells able to re-start replication. This population was clearly reduced in POLD1- or POLD3-depleted cells, suggesting a role for both Pol δ subunits in stabilization and restart of stalled RFs. As expected, CPT-treated cells were not affected, since this drug induces DNA damage in a replication-dependent manner whereas we are only analyzing cells treated in G1 that enter S-phase. The importance of POLD1 and POLD3 in replication after genotoxic stress was supported by HU and CPT sensitivity of cells depleted of these proteins, as evaluated by a clonogenic assay (Supplementary Fig. S4).

### γH2AX foci accumulation is rescued by RNase H1 overexpression in POLD3-depleted cells

**Figure 5:**
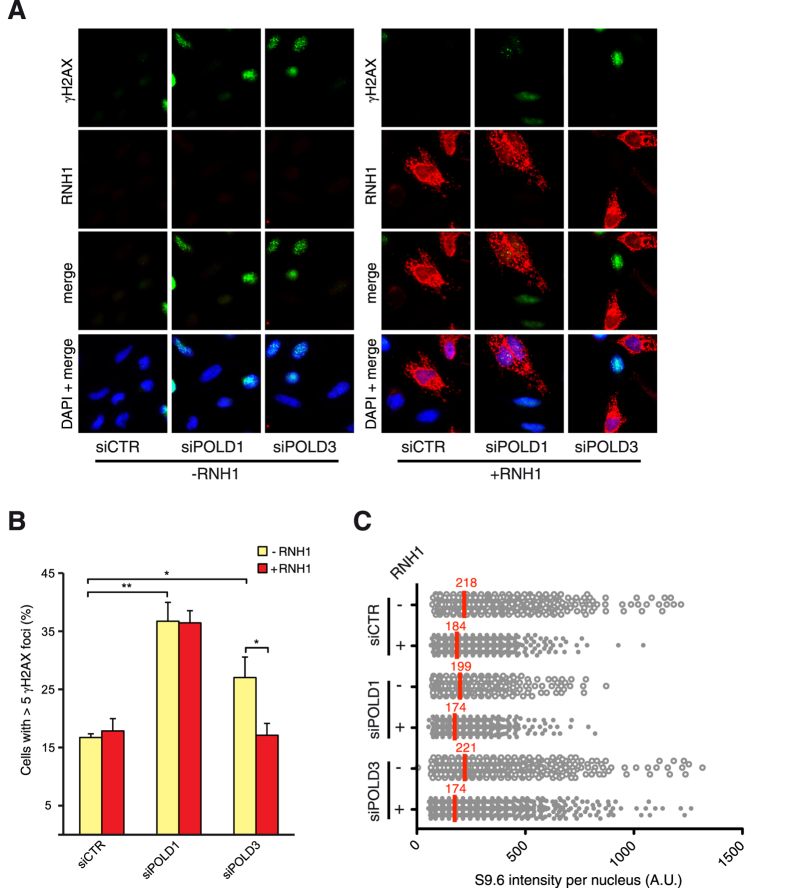
RNA-DNA hybrids-dependent γH2AX foci and S9.6 signal in POLD1- and POLD3-depleted cells. (**A**) Immunofluorescence of HeLa cells stained with antibodies against γH2AX and RNase H1. HeLa cells were transfected with pcDNA3 (-RNH1) or pcDNA3-RNaseH1 (+RNH1) for RNase H1 overexpression. (**B**) Percentage of cells with more than 5 foci. More than 100 cells overexpressing RNase H1 (positive-stained) or more than 100 cells of mixed population transfected with the empty vector were counted in each experiment. Means ± SEM from at least three independent experiments are shown. Differences between distributions were assessed by the Mann-Whitney test. (**C**) Immunofluorescence of HeLa cells stained with antibodies against S9.6. The graph shows the median of the S9.6 signal intensity per nucleus after nucleolar signal removal in siCTR, siPOLD1 and siPOLD3 HeLa cells ± RNase H1 overexpression. More than 1000 cells from at least five independent experiments were considered, adjusting the intensity values to the average of the medians.

There is mounting evidence that RNA-DNA hybrids are a common natural source of replication stress^35^, thereafter we evaluated the impact of RNA-DNA hybrids on the genome instability associated with the depletion of POLD1 and POLD3. To this extent, after siRNA-mediated depletion of POLD1 or POLD3, we transfected cells with a plasmid overexpressing the human RNase H1 or with the empty vector and the percentage of cells containing more than 5 γH2AX foci were scored (Fig. 5A and B). Interestingly the overexpression of RNase H1 abolished the increase of cells with γH2AX foci caused by siPOLD3 but not the increase caused by siPOLD1. Neither POLD3 nor POLD1 knockdown caused accumulation of RNA-DNA hybrids as assessed by immunofluorescence with S9.6 antibodies (Fig. 5C). These results suggest that replication stress occurring in POLD3-depleted cells is largely caused by RNA-DNA hybrids, attributing a specific role of POLD3 in the repair of RNA-DNA hybrids-mediated DNA breaks or replication restart of forks stalled at RNA-DNA hybrids. Accumulation of γH2AX foci after POLD3 depletion was rescued by transfection with a plasmid overexpressing POLD3, demonstrating that the DNA damage caused by siRNA-mediated POLD3-depletion was specifically dependent on the reduction of POLD3 levels and not due to off-target effects (Supplementary Fig. S5).

### Pol ζ role in RNA-DNA hybrid-dependent DNA damage

**Figure 6:**
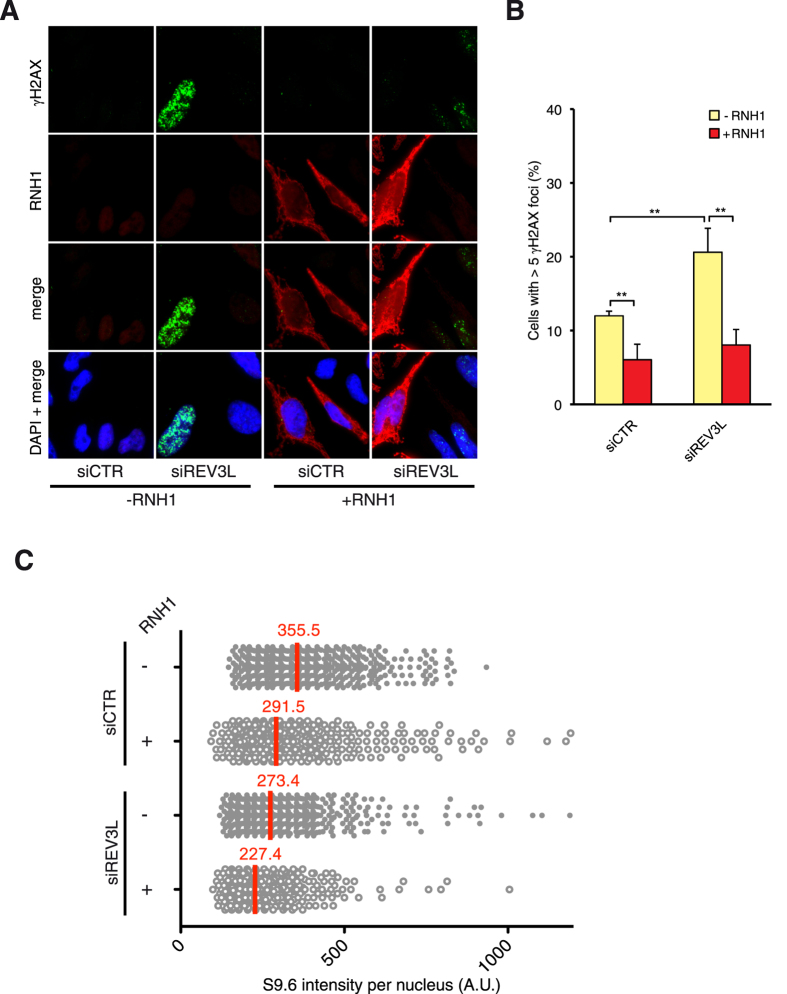
γH2AX foci and S9.6 signal in REV3L-depleted cells overexpressing RNase H1. (**A**) Immunofluorescence of HeLa cells stained with antibodies against γH2AX and RNase H1. HeLa cells were transfected with pcDNA3 (−RNH1) or pcDNA3-RNaseH1 (+RNH1) for RNase H1 overexpression. (**B**) Percentage of cells with more than 5 foci. More than 100 cells overexpressing RNase H1 (positive-stained) or more than 100 cells of mixed population transfected with the empty vector were counted in each experiment. Means ± SEM from three independent experiments are shown. Differences between distributions were assessed by the one-tailed Mann-Whitney test. (**C**) Immunofluorescence of HeLa cells stained with antibodies against S9.6. The graph shows the median of the S9.6 signal intensity per nucleus after nucleolar signal removal in siCTR and siREV3L HeLa cells ± RNase H1 overexpression. More than 400 cells from three independent experiments were considered, adjusting the intensity values to the average of the medians.

Given that POLD3 together with POLD2 could work either in the Pol δ replicative complex or in the translesion synthesis Pol ζ complex and that the RNA-DNA hybrid-dependent genome instability is specific of POLD3 but not of POLD1 depletion, we wondered whether this phenotype could be associated with the Pol ζ complex. After siRNA-mediated depletion of the Pol ζ catalytic subunit REV3L, we found an increase in γH2AX foci as already reported^36^ (Fig. 6A and B). Interestingly, after transfection with a plasmid overexpressing the human RNase H1, γH2AX foci level significantly decreased, suggesting that Pol ζ together with POLD3 has a role in RNA-DNA hybrid-dependent genome instability. The expression levels of POLD3 mRNA were not affected by the depletion of REV3L and vice versa, so that we can rule out that these phenotypes could be due to destabilization of one protein in the absence of the other (Supplementary Fig. S6). Again, as observed with POLD3 depletion, REV3L depletion did not lead to a detectable accumulation of RNA-DNA hybrids (Fig. 6C).

## Discussion

Perturbation of DNA replication is often linked to the emergence of genome instability, a condition associated with tumorigenesis as well as aging. Here we describe the effects on genome integrity caused by the depletion of POLD1 and POLD3, two subunits of Pol δ, one of the main replicative polymerases together with Pol ε and the Pol α-primase complex^4^. We clearly showed that the downregulation of each of these subunits not only give rise to shared genome instability hallmarks, but also to different phenotypes, pointing toward the existence of distinct events controlled by the different subunits of Pol δ.

*POLD1* or *POLD3* knockdown impaired cell proliferation (Supplementary Fig. S1E), but while depletion of the catalytic subunit POLD1 caused a more severe phenotype, POLD3 depletion caused only a mild effect. This is consistent with POLD3 not being essential for RF progression, as is the case of the Pol32 budding yeast counterpart^12^. Importantly, however, POLD3 is essential in *S. pombe*^21^ and mammals, provided the recent report showing that *POLD3* knock out leads to embryonic lethality in mic^37^. Certainly, the phenotypes described here must be a consequence of a great reduction rather than a complete ablation of the protein after siRNA knockdown. Since POLD1 is essential for replication it cannot be removed completely from the cell without producing cell cycle arrest and cell death.

POLD1-depleted cells released from G1/S-phase synchronization were delayed in entering and completing S-phase accordingly with the essential function of DNA Pol δ (Fig. 1C,D and Supplementary Fig. S1B–D) and as already shown for POLD1-depletion^38^. Moreover a fraction of this cell population was arrested in G2 as manifested by the higher amount of G2-phase cells that escaped the cell cycle synchronization (Supplementary Fig. S1A). Likely, this was a consequence of the high levels of DNA damage, detected by flow cytometry as γH2AX positive cells in G2-phase after POLD1 depletion (Fig. 2G). On the other hand POLD3-depleted cells entered and progressed into S-phase with minor difficulties, but were delayed at the end of this phase (Fig. 1C,D and Supplementary Fig. S1B–D). The longer time required to complete the S-phase could be caused by an accumulation of DNA damage during replication, possibly due to the incapacity of these cells to complete replication at difficult-to-replicate regions because of secondary structures, transcription or heterochromatin^39^. Indeed, the accumulation of γH2AX was mainly associated with S-phase both after POLD1 and POLD3 depletion, as detected by immunofluorescence and flow cytometry (Fig. 2C,D and G).

Notably, in both cases we detected an increase in interorigin distance by DNA combing (Fig. 1G), consistent with a role of Pol δ at replication origins. Indeed, POLD1 has been previously shown using chromatin immunoprecipitation to bind to origins in G1/S-phase synchronized HeLa cells^40^. A decrease in origin density could result in cells reaching mitosis without having completed replication that in turn could lead to DNA breaks, sister chromatid recombination and chromosome abnormalities. Indeed scarcity of active replication origins has been shown to be associated with chromosome instability at common fragile sites^41^,^42^.

Remarkably, at molecular level, the replication fork velocity was not affected by the depletion of Pol δ subunits (Fig. 1F). This could be explained with the capability of DNA combing to detect only active RFs, hence replication tracks generated by a polymerase engaged in replication and not targeted by siRNA silencing. Interestingly, POLD1-depletion has been shown to cause a reduction of the replication fork velocity^38^. The reason for such apparent discrepancy could be a different degree of *POLD1* knockdown. Indeed if Pol δ was completely removed from the cells, incorporation of nucleotide analogs would not be possible and no labeled fibers would be visible. Only fibers with a sufficient amount of Pol δ to undertake replication would be detectable.

Cells depleted of POLD1 or POLD3 accumulated more γH2AX and 53BP1 foci than control cells, the increase of γH2AX foci being much higher in POLD1-depleted cells whereas 53BP1 foci were similarly increased in both POLD1 and POLD3-depleted cells (Fig. 2A and B). It is possible that POLD1-depletion causes different kinds of damage, some shared with POLD3 depletion and others not. POLD3 depletion showed several phenotypes but we could not exclude that some of them could be partially dependent on POLD1. Importantly, the accumulation of γH2AX foci was specifically abolished by RNase H1 overexpression only in POLD3-depleted cells but not after POLD1 depletion (Fig. 5A and B). This latter phenotype could be partly related to the role of POLD3 as part of the Pol ζ complex since REV3L depletion conferred similar RNA-DNA hybrid-dependent genome instability (Fig. 6).

Additionally, the fact that POLD3 also acts in BIR, suggest that this repair pathway may also prevent genome instability generated by hybrids. Interestingly, the increase of RNA-DNA hybrid-dependent DNA damage was not accompanied by an increase in RNA-DNA hybrids as shown by S9.6 immunofluorescence in either POLD3 or REV3L depletion (Figs 5C and 6C). This phenotype can be explained by the fact that RNA-DNA hybrids are formed in normal cells^43^ and that DNA repair mechanisms including BRCA and FA pathways are required to prevent their DNA damaging effects^44^,^45^,^46^,^47^. Proper repair involving POLD3 and REV3L may be required for replication or repair to bypass the hybrids as well as other DNA damages, so that in their absence DNA breaks would accumulate without generating *de novo* hybrids. The situation is different from cells depleted of mRNP biogenesis factors, such as the THO complex, that lead to an increase of hybrid formation above the WT levels^48^. In any case, we cannot discard at this point whether RNase H1 overexpression rescued at least part of the increase in DNA damage by clearing RNA-DNA hybrids putatively formed as replication or DSB repair intermediates due to the hypomorphic activity of Pol δ after POLD3 depletion.

Our results suggest that the role of POLD3 in RNA-DNA hybrid-mediated genome instability would be at least partially exerted as a component of the Pol ζ complex rather than of the Pol δ complex. The recent observation that replication-associated DNA repair pathways, such as Fanconi Anemia^45^,^47^, are crucial for preventing RNA-DNA hybrid accumulation, prompt us to suggest that Pol ζ-associated POLD3 might be a key player in the replicative repair of damage generated by naturally-occurring RNA-DNA hybrid. Indeed REV3L has been shown to be an important player in ICL repair^49^,^50^ and furthermore the FA pathway is critical for efficient Pol ζ-dependent TLS error prone repair^51^. A physical analysis of replication stress generated by RNA-DNA hybrids in POLD3- and REV3L-depleted cells would be required to better understand the impact of RNA-DNA hybrid in cell homeostasis and genome integrity, and the mechanisms by which RNA-DNA hybrid-mediated DNA damage is repaired via POLD3. In this sense, it is also possible that part of the effect observed in POLD3-depleted cells in RNA-DNA hybrid-mediated damage might also be related to its DSB repair function.

The presence of γH2AX foci but not that of 53BP1 foci was mainly associated with the S-phase (Fig. 2C and D), suggesting that the damage is generated during DNA replication. Importantly, being the 53BP1 foci phenotype similar after POLD1 or POLD3 depletion, we could rule out that the milder phenotype observed in other experiments were due to lower efficiency of *POLD3* knockdown and a consequent incomplete penetrance of phenotypes.

Depletion of POLD1 or POLD3 caused also a general increase in SCEs and chromosomes segregation defects (Fig. 3). This could be due to increased DNA damage or to perturbation of the balance between the different HR types of events during repair. POLD3 depletion induced specifically more anaphase bridges than POLD1 depletion, while the difference between them was the opposite for lagging chromosomes, POLD1 depletion being associated with a slightly higher amount of lagging chromosomes. The increase in anaphase bridges in POLD3-depleted cells could be due to telomere instability, since the POLD3 yeast homolog Pol32 is needed for telomere homeostasis mediated by BIR^22^. Indeed, a consequence of telomere erosion in human cells could be the ligation of broken chromosome ends in “breakage-fusion-bridge” events^52^. POLD3-dependent BIR has also been recently shown to occur in human cells using a GFP-based reporter system, in which the sequence homology was restricted to one side of a DSB induced by the nuclease I-*Sce*I^23^. It is thus possible that the difference observed in anaphase bridges (Fig. 3C) might be caused by the specific role that POLD3 has in the repair of damaged forks by BIR. Alternatively, the increase of chromosome instability in POLD3-depleted cells could be a consequence of its recently described function of driving DNA synthesis during mitosis of under-replicated chromosome regions^26^. Consistent with the observation that POLD3-depletion induces replication failure and segmental genomic duplication following cyclin E-induced replicative stress in human cell lines^23^, POLD3- and POLD1-depleted cells had more difficulties overcoming replicative stress and DNA damage (Fig. 4). In agreement with this conclusion, it has just been reported that POLD3-depletion also induces replication stress and DNA damage in mice^37^.

Interestingly, the two subunits of the TLS polymerase Pol ζ (REV3L and REV7) have been recently shown to form a functional complex with the accessory Pol δ subunits POLD2 and POLD3^14^, and REV3L is required to prevent CFS expression^20^. Notably, REV3L depletion also causes a specific increase of anaphase bridges while REV7 does not. Instead it causes a specific increase of lagging chromosomes. Anaphase bridges in POLD3-depleted cells could be due to a loss of POLD3 interaction with Pol ζ and, thus, to a specific defect in post-replicative repair. Either by a defect in BIR or TLS, the results suggest that POLD3 has an additional function over POLD1 in genome stability. Moreover POLD3 exerts an important role in translesion DNA synthesis, which it promotes independently of DNA polymerase ζ^53^.

In addition to the spontaneous accumulation of DNA damage and chromosomes abnormalities, we also showed that POLD1 and POLD3 are important for S-phase progression under replicative stress and damaging conditions (Fig. 4). Indeed the amount of cells that stop synthesizing DNA upon CPT treatment, a DNA topoisomerase I inhibitor that induces DSBs in a replication-dependent manner^54^, increased both in POLD1- and POLD3-depleted cells, suggesting an important role of both subunits in overcoming DNA damage during replication. Furthermore, these proteins also showed a role in replication fork stabilization since the percentage of cells unable to resume replication and S-phase progression increased following treatment with HU, which causes RF stalling but not collapse^55^

Our work, therefore, establishes that POLD1 and POLD3 work together but with a differential role in genome integrity. They are important in unperturbed conditions to prevent genome instability, and after replicative stress or induced DNA damage to preserve replication forks stability. In addition, we found that they are needed for efficient S-phase progression and to maintain the proper number of active replication origins. In their absence the impairment of S-phase progression and the scarcity of active origins could help to generate genome instability resulting in DSBs and chromosome segregation defects as a consequence of incomplete chromosome duplication. Moreover, the specific increase of anaphase bridges of POLD3-depleted cells as well as other phenotypes suggests that POLD3 has an additional function to that of Pol δ in DNA metabolism, including BIR or post-replicative TLS, in part occurring at RNA-DNA hybrid-forming regions, a function that might be associated with the Pol ζ complex.

## Materials and Methods

### Cell culture and treatments

HeLa, U2OS, HEK 293 T and MCF7-DRGFP cells were maintained in DMEM medium, supplemented with 10% heat-inactivated fetal calf serum (FCS) and cultured at 37 °C in a humidified atmosphere containing 5% CO_2_. HeLa cells synchronization at G1/S phase transition was obtained by double thymidine block started at 72 h after the first siRNA transfection. Exponentially growing cells were treated for 19 hours with 2 mM thymidine in growth medium, washed twice with PBS, released in fresh medium for 7 hours and then treated again for 17 hours with 2 mM thymidine. Synchronized cells were washed twice with PBS and released into fresh medium. Cell cycle was analyzed at different time points after release. Before harvest cells were pulse-labeled with EdU 20 μM for 20 minutes to label S-phase cells.

### Antibodies

For Western blot analysis, antibodies anti-POLD1 (sc-17776), anti-POLD3 (H00010714-M01) and anti-β-Actin (Ab8226) were purchased from Santa Cruz Biotechnology, Abnova and Abcam, respectively. For immunofluorescence, antibodies anti-γH2AX (JBW301) and anti-53BP1 (NB100-304) were purchased from Upstate and Novus Biologicals, respectively. The S9.6 antibody was purified from the hybridoma cell line HB-8730.

### siRNA transfection

Cells were reverse-transfected twice with siRNAs at the final concentration of 75 nM using Lipofectamine 2000 (Invitrogen). The second transfection was performed 48 h after the first one, and all assays were performed 48 hours after the second transfection unless otherwise specified. A mixture of 4 individual siRNA (ON-TARGETplus SMARTpool *siRNA)* targeting *POLD3, POLD1* or *REV3L* and a non-targeting pool control were obtained from Dharmacon. For *ATR* knockdown, the previously published oligonucleotide 5′-AAC GAG ACU UCU GCG GAU UGC-3′ 56 was obtained from MWG. siRNA efficiency was checked by immunoblot assay.

### Immunofluorescence

Previously siRNA transfected HeLa cells seeded on glass coverslips were fixed with 3.7% formaldehyde in PBS for 15 minutes, washed 4 times with PBS, permeabilized with 0.5% Triton X-100 in PBS for 5 minutes and blocked with 3% bovine serum albumin (BSA) in PBS for 1 hour. The coverslips were then incubated with primary antibodies diluted 1:500 in 3% BSA in PBS for 2 hours followed by 3 washes with PBS and 1 hour incubation with 1:1000 diluted secondary antibodies conjugated with Alexa Fluor 488 (goat anti-mouse) and Alexa Fluor 568 (goat anti-rabbit) (Invitrogen). After 2 washes with PBS, nuclei were counterstained with 10 μg/ml DAPI in PBS for 5 minutes, washed 3 more times and mounted with ProLong Gold antifade reagent (Invitrogen). For the EdU labeling, cells were pulse-labeled with 10 μM EdU for 20 minutes before fixation. EdU staining was performed with a Click-iT EdU Alexa Fluor 488 Imaging kit (Invitrogen) according to manufacturer’s instructions. Random images were acquired with a 63X objective and foci were scored using the MetaMorph software. Micronuclei were manually scored at microscope using DAPI staining. At least 100 cells were scored for each condition.

S9.6 immunofluorescence was performed as previously described^57^ incubating with 1:500 diluted S9.6 antibody.

### FACS analysis

For the concomitant staining of EdU and BrdU, cells were first pulse-labeled with EdU 20 μM added directly to the growing medium. After 30 minutes, hydroxyurea (HU, Sigma, 4 mM for 6 hours) or camptothecin (CPT, Sigma, 2.5 μM for 1 hour) was added to the medium or alternatively cells were left untreated. Following the treatment cells were washed with PBS and released in medium containing BrdU 10 μM for 20 minutes. Cells were harvested, fixed with 70% ethanol in PBS and incubated on ice for 1 hour. Cells were treated with 2 N HCl 0.5% Triton X-100 for 30 minutes at room temperature, treated with 0.1 M Sodium tetraborate pH 8.5, washed once with washing buffer (1% BSA 0.1% Triton X-100 in PBS) and stained with a Click-iT EdU Alexa Fluor 488 Imaging kit (Invitrogen) according to manufacturer’s instructions. After 3 washes with washing buffer, cells were incubated for 30 minutes in the same buffer containing 1:25 anti-BrdU antibody conjugated with Alexa Fluor 647 (B35140, Invitrogen) and 0.5 μg/μl RNase A, washed once and resuspended in PBS containing 7AAD (51-68981E, BD) diluted 1:50 to counterstain DNA. After 30 minutes cells were examined by flow cytometry (FACSCalibur; BD).

For the labeling with only one nucleotide analogue, EdU labeling was preferred. Cells were stained with a Click-iT EdU Alexa Fluor 488 Imaging kit (Invitrogen) according to manufacturer’s instructions. After 3 washes with washing buffer (1% BSA 0.1% Triton X-100 in PBS), DNA was counterstained with 7AAD (51-68981E, BD) diluted 1:50 and 0.5 μg/μl RNase A in PBS. After 30 minutes cells were examined by flow cytometry (FACSCalibur; BD).

For the concomitant staining of γH2AX and EdU, cells were pulse-labeled with EdU 10 μM for 20 minutes before harvest and fixation. Cells were treated with 0.5% Triton X-100 for 5 minutes followed by EdU staining with Click-iT EdU Alexa Fluor 488 Imaging kit (Invitrogen) according to manufacturer’s instructions. After 3 washes with washing buffer (1% BSA 0.1% Triton X-100 in PBS), cells were incubated for 1 hour in the same buffer containing 1:100 anti-γH2AX antibody and 0.5 μg/μl RNase A. Following one wash with washing buffer, cells were incubated with secondary antibodies conjugated with Alexa Fluor 647 (goat anti-mouse) (Invitrogen) diluted 1:100 in the same buffer for 30 minutes, washed once and DNA was counterstained with 7AAD (51-68981E, BD) diluted 1:50 in PBS. After 30 minutes cells were examined by flow cytometry (FACSCalibur; BD). CellQuest software was used for acquisition and analysis of data.

### DNA combing

DNA combing was performed as previously described^58^. Cells were labeled and harvested 96 hours after the first siRNA transfection. Briefly, DNA fibers were extracted from cells in agarose plugs immediately after CldU labeling and were stretched on silanized coverslips. DNA molecules were counterstained with an anti-ssDNA antibody (MAB3868, Chemicon; 1/100) and an anti-mouse IgG coupled to Alexa 647 (A21241, Molecular Probes, 1/50). CldU and IdU were detected with BU1/75 (AbCys, 1/20) and an anti-rat IgG coupled to Alexa 488 (A21470, Molecular Probes, 1/50) or B44 (Becton Dickinson, 1/20) anti-BrdU antibodies and an anti-mouse IgG coupled to Alexa 546 (A21123, Molecular Probes, 1/50), respectively. DNA fibers were analyzed on a Leica DM6000 microscope equipped with a DFC390 camera (Leica). Data acquisition was performed with LAS AF (Leica). The values of fork velocity and interorigin distance were calculated as previously described^59^.

### Chromosome abnormalities

To score for anaphase bridges or lagging chromosome, HeLa cells were seeded on glass coverslips, treated with 50 ng/ml Nocodazole (Sigma) for 4 hours, washed 3 times with PBS and release in complete medium for 1 hour. Cells were fixed and permeabilized by the addition of 1 volume of PBS/Formaldehyde/Triton X-100 to get the final concentration of 3.7% Formaldehyde and 0.25% Triton X-100. After 20 minute the coverslips were gently removed from the well, quickly rinsed in distilled water and mounted in Vectashield mounting medium with DAPI (H-1200, Vector Laboratories). At least 50 anaphases were scored for each condition.

### SCE

SCE assay was performed as previously described^60^. Briefly, HeLa or U2OS cells were incubated with 10 μM BrdU for 42 hours followed by 3 hours treatment with 0.1 μg/ml of KaryoMAX colcemid solution (Invitrogen). Cells were resuspended in 0.075 M KCl and incubated at 37 °C for 10 minutes followed by 3 changes of Carnoy fixative (3:1 methanol:acetic acid). Cells were dropped onto slides and baked overnight at 65 °C. To differentially stain the two chromatids, the slides were incubated with 20 μg/ml Hoechst solution for 20 minutes, then exposed for 1 hour to UVA irradiation in SSC 2X and incubated in SSC 2X at 60 °C for 20 minutes before standard Giemsa staining was performed. Metaphases were scored using a 100X objective.

### Statistical analysis

Statistical significance was determined by paired t-tests or the Mann-Whitney test as specified. All tests performed were 2-tailed unless otherwise specified and three levels of statistical significance were considered: *p ≤ 0.05, **p ≤ 0.01 and ***p ≤ 0.001.

### Data availability statement

All relevant data supporting this study are provided in full in the results section or as supplementary information accompanying this paper.

## Additional Information

**How to cite this article**: Tumini, E. *et al*. Roles of human POLD1 and POLD3 in genome stability. *Sci. Rep.* **6**, 38873; doi: 10.1038/srep38873 (2016).

**Publisher's note:** Springer Nature remains neutral with regard to jurisdictional claims in published maps and institutional affiliations.

## Supplementary Material

Supplementary Information

## References

[b1] Prindle M. J. & Loeb L. A. DNA polymerase delta in DNA replication and genome maintenance. Environ Mol Mutagen 53, 666–682 (2012). 23065663 10.1002/em.21745 PMC3694620

[b2] Georgescu R. E. . Reconstitution of a eukaryotic replisome reveals suppression mechanisms that define leading/lagging strand operation. Elife 4, e04988 (2015). 25871847 10.7554/eLife.04988 PMC4413876

[b3] Nick McElhinny S. A., Gordenin D. A., Stith C. M., Burgers P. M. & Kunkel T. A. Division of labor at the eukaryotic replication fork. Mol Cell 30, 137–144 (2008). 18439893 10.1016/j.molcel.2008.02.022 PMC2654179

[b4] Hubscher U., Maga G. & Spadari S. Eukaryotic DNA polymerases. Annu Rev Biochem 71, 133–163 (2002). 12045093 10.1146/annurev.biochem.71.090501.150041

[b5] Lemoine F. J., Degtyareva N. P., Kokoska R. J. & Petes T. D. Reduced levels of DNA polymerase delta induce chromosome fragile site instability in yeast. Mol Cell Biol 28, 5359–5368 (2008). 18591249 10.1128/MCB.02084-07 PMC2519721

[b6] Huang Q. M. . Regulation of DNA polymerase POLD4 influences genomic instability in lung cancer. Cancer Res 70, 8407–8416 (2010). 20861182 10.1158/0008-5472.CAN-10-0784

[b7] Lee M. Y. . The tail that wags the dog: p12, the smallest subunit of DNA polymerase delta, is degraded by ubiquitin ligases in response to DNA damage and during cell cycle progression. Cell Cycle 13, 23–31 (2014). 24300032 10.4161/cc.27407 PMC3925730

[b8] Lovejoy C. A. . Functional genomic screens identify CINP as a genome maintenance protein. Proc Natl Acad Sci USA 106, 19304–19309 (2009). 19889979 10.1073/pnas.0909345106 PMC2780779

[b9] Paulsen R. D. . A genome-wide siRNA screen reveals diverse cellular processes and pathways that mediate genome stability. Mol Cell 35, 228–239 (2009). 19647519 10.1016/j.molcel.2009.06.021 PMC2772893

[b10] Song J. . Human POLD1 modulates cell cycle progression and DNA damage repair. BMC Biochem 16, 14 (2015). 26087769 10.1186/s12858-015-0044-7 PMC4471906

[b11] Rahmeh A. A. . Phosphorylation of the p68 subunit of Pol delta acts as a molecular switch to regulate its interaction with PCNA. Biochemistry 51, 416–424 (2012). 22148433 10.1021/bi201638e

[b12] Gerik K. J., Li X., Pautz A. & Burgers P. M. Characterization of the two small subunits of Saccharomyces cerevisiae DNA polymerase delta. J Biol Chem 273, 19747–19755 (1998). 9677405 10.1074/jbc.273.31.19747

[b13] Hanna M., Ball L. G., Tong A. H., Boone C. & Xiao W. Pol32 is required for Pol zeta-dependent translesion synthesis and prevents double-strand breaks at the replication fork. Mutat Res 625, 164–176 (2007). 17681555 10.1016/j.mrfmmm.2007.06.008

[b14] Baranovskiy A. G. . DNA polymerase delta and zeta switch by sharing accessory subunits of DNA polymerase delta. J Biol Chem 287, 17281–17287 (2012). 22465957 10.1074/jbc.M112.351122 PMC3366816

[b15] Johnson R. E., Prakash L. & Prakash S. Pol31 and Pol32 subunits of yeast DNA polymerase delta are also essential subunits of DNA polymerase zeta. Proc Natl Acad Sci USA 109, 12455–12460 (2012). 22711820 10.1073/pnas.1206052109 PMC3411960

[b16] Lee Y. S., Gregory M. T. & Yang W. Human Pol zeta purified with accessory subunits is active in translesion DNA synthesis and complements Pol eta in cisplatin bypass. Proc Natl Acad Sci USA 111, 2954–2959 (2014). 24449906 10.1073/pnas.1324001111 PMC3939873

[b17] Makarova A. V., Stodola J. L. & Burgers P. M. A four-subunit DNA polymerase zeta complex containing Pol delta accessory subunits is essential for PCNA-mediated mutagenesis. Nucleic Acids Res 40, 11618–11626 (2012). 23066099 10.1093/nar/gks948 PMC3526297

[b18] Makarova A. V. & Burgers P. M. Eukaryotic DNA polymerase zeta. DNA Repair (Amst) 29, 47–55 (2015). 25737057 10.1016/j.dnarep.2015.02.012 PMC4426032

[b19] Gan G. N., Wittschieben J. P., Wittschieben B. O. & Wood R. D. DNA polymerase zeta (pol zeta) in higher eukaryotes. Cell Res 18, 174–183 (2008). 18157155 10.1038/cr.2007.117

[b20] Bhat A., Andersen P. L., Qin Z. & Xiao W. Rev3, the catalytic subunit of Polzeta, is required for maintaining fragile site stability in human cells. Nucleic Acids Res 41, 2328–2339 (2013). 23303771 10.1093/nar/gks1442 PMC3575803

[b21] MacNeill S. A., Moreno S., Reynolds N., Nurse P. & Fantes P. A. The fission yeast Cdc1 protein, a homologue of the small subunit of DNA polymerase delta, binds to Pol3 and Cdc27. EMBO J 15, 4613–4628 (1996). 8887553 PMC452193

[b22] Lydeard J. R., Jain S., Yamaguchi M. & Haber J. E. Break-induced replication and telomerase-independent telomere maintenance require Pol32. Nature 448, 820–823 (2007). 17671506 10.1038/nature06047

[b23] Costantino L. . Break-induced replication repair of damaged forks induces genomic duplications in human cells. Science 343, 88–91 (2014). 24310611 10.1126/science.1243211 PMC4047655

[b24] Malkova A. & Ira G. Break-induced replication: functions and molecular mechanism. Curr Opin Genet Dev 23, 271–279 (2013). 23790415 10.1016/j.gde.2013.05.007 PMC3915057

[b25] Pardo B. & Aguilera A. Complex chromosomal rearrangements mediated by break-induced replication involve structure-selective endonucleases. PLoS Genet 8, e1002979 (2012). 23071463 10.1371/journal.pgen.1002979 PMC3459980

[b26] Minocherhomji S. . Replication stress activates DNA repair synthesis in mitosis. Nature 528, 286–290 (2015). 26633632 10.1038/nature16139

[b27] Dunlop M. G. . Common variation near CDKN1A, POLD3 and SHROOM2 influences colorectal cancer risk. Nat Genet 44, 770–776 (2012). 22634755 10.1038/ng.2293 PMC4747430

[b28] Rayner E. . A panoply of errors: polymerase proofreading domain mutations in cancer. Nat Rev Cancer 16, 71–81 (2016). 26822575 10.1038/nrc.2015.12

[b29] Spier I. . Frequency and phenotypic spectrum of germline mutations in POLE and seven other polymerase genes in 266 patients with colorectal adenomas and carcinomas. Int J Cancer 137, 320–331 (2015). 25529843 10.1002/ijc.29396

[b30] Valle L. . New insights into POLE and POLD1 germline mutations in familial colorectal cancer and polyposis. Hum Mol Genet 23, 3506–3512 (2014). 24501277 10.1093/hmg/ddu058

[b31] Boulet A., Simon M., Faye G., Bauer G. A. & Burgers P. M. Structure and function of the Saccharomyces cerevisiae CDC2 gene encoding the large subunit of DNA polymerase III. EMBO J 8, 1849–1854 (1989). 2670563 10.1002/j.1460-2075.1989.tb03580.x PMC401032

[b32] Aguilera A. & Garcia-Muse T. Causes of genome instability. Annu Rev Genet 47, 1–32 (2013). 23909437 10.1146/annurev-genet-111212-133232

[b33] Thompson S. L., Bakhoum S. F. & Compton D. A. Mechanisms of chromosomal instability. Curr Biol 20, R285–295 (2010). 20334839 10.1016/j.cub.2010.01.034 PMC3781365

[b34] Pierce A. J., Johnson R. D., Thompson L. H. & Jasin M. XRCC3 promotes homology-directed repair of DNA damage in mammalian cells. Genes Dev 13, 2633–2638 (1999). 10541549 10.1101/gad.13.20.2633 PMC317094

[b35] Santos-Pereira J. M. & Aguilera A. R loops: new modulators of genome dynamics and function. Nat Rev Genet 16, 583–597 (2015). 26370899 10.1038/nrg3961

[b36] Knobel P. A., Kotov I. N., Felley-Bosco E., Stahel R. A. & Marti T. M. Inhibition of REV3 expression induces persistent DNA damage and growth arrest in cancer cells. Neoplasia 13, 961–970 (2011). 22028621 10.1593/neo.11828 PMC3201572

[b37] Murga M. . POLD3 Is Haploinsufficient for DNA Replication in Mice. Mol Cell 63, 877–883 (2016). 27524497 10.1016/j.molcel.2016.07.007 PMC5029548

[b38] Bermudez V. P., Farina A., Raghavan V., Tappin I. & Hurwitz J. Studies on human DNA polymerase epsilon and GINS complex and their role in DNA replication. J Biol Chem 286, 28963–28977 (2011). 21705323 10.1074/jbc.M111.256289 PMC3190704

[b39] Mirkin E. V. & Mirkin S. M. Replication fork stalling at natural impediments. Microbiol Mol Biol Rev 71, 13–35 (2007). 17347517 10.1128/MMBR.00030-06 PMC1847372

[b40] Vaara M. . Segregation of replicative DNA polymerases during S phase: DNA polymerase epsilon, but not DNA polymerases alpha/delta, are associated with lamins throughout S phase in human cells. J Biol Chem 287, 33327–33338 (2012). 22887995 10.1074/jbc.M112.357996 PMC3460436

[b41] Ozeri-Galai E. . Failure of origin activation in response to fork stalling leads to chromosomal instability at fragile sites. Mol Cell 43, 122–131 (2011). 21726815 10.1016/j.molcel.2011.05.019

[b42] Letessier A. . Cell-type-specific replication initiation programs set fragility of the FRA3B fragile site. Nature 470, 120–123 (2011). 21258320 10.1038/nature09745

[b43] Ginno P. A., Lott P. L., Christensen H. C., Korf I. & Chedin F. R-loop formation is a distinctive characteristic of unmethylated human CpG island promoters. Mol Cell 45, 814–825 (2012). 22387027 10.1016/j.molcel.2012.01.017 PMC3319272

[b44] Bhatia V. . BRCA2 prevents R-loop accumulation and associates with TREX-2 mRNA export factor PCID2. Nature 511, 362–365 (2014). 24896180 10.1038/nature13374

[b45] Garcia-Rubio M. L. . The Fanconi Anemia Pathway Protects Genome Integrity from R-loops. PLoS Genet 11, e1005674 (2015). 26584049 10.1371/journal.pgen.1005674 PMC4652862

[b46] Hatchi E. . BRCA1 recruitment to transcriptional pause sites is required for R-loop-driven DNA damage repair. Mol Cell 57, 636–647 (2015). 25699710 10.1016/j.molcel.2015.01.011 PMC4351672

[b47] Schwab R. A. . The Fanconi Anemia Pathway Maintains Genome Stability by Coordinating Replication and Transcription. Mol Cell 60, 351–361 (2015). 26593718 10.1016/j.molcel.2015.09.012 PMC4644232

[b48] Castellano-Pozo M. . R loops are linked to histone H3 S10 phosphorylation and chromatin condensation. Mol Cell 52, 583–590 (2013). 24211264 10.1016/j.molcel.2013.10.006

[b49] Zhang N., Liu X., Li L. & Legerski R. Double-strand breaks induce homologous recombinational repair of interstrand cross-links via cooperation of MSH2, ERCC1-XPF, REV3, and the Fanconi anemia pathway. DNA Repair (Amst) 6, 1670–1678 (2007). 17669695 10.1016/j.dnarep.2007.06.002 PMC2586762

[b50] Shen X. . REV3 and REV1 play major roles in recombination-independent repair of DNA interstrand cross-links mediated by monoubiquitinated proliferating cell nuclear antigen (PCNA). J Biol Chem 281, 13869–13872 (2006). 16571727 10.1074/jbc.C600071200

[b51] Niedzwiedz W. . The Fanconi anaemia gene FANCC promotes homologous recombination and error-prone DNA repair. Mol Cell 15, 607–620 (2004). 15327776 10.1016/j.molcel.2004.08.009

[b52] Bunting S. F. & Nussenzweig A. End-joining, translocations and cancer. Nat Rev Cancer 13, 443–454 (2013). 23760025 10.1038/nrc3537 PMC5724777

[b53] Hirota K. . The POLD3 subunit of DNA polymerase delta can promote translesion synthesis independently of DNA polymerase zeta. Nucleic Acids Res 43, 1671–1683 (2015). 25628356 10.1093/nar/gkv023 PMC4330384

[b54] Yamauchi T., Yoshida A. & Ueda T. Camptothecin induces DNA strand breaks and is cytotoxic in stimulated normal lymphocytes. Oncol Rep 25, 347–352 (2011). 21165573 10.3892/or.2010.1100

[b55] Petermann E., Orta M. L., Issaeva N., Schultz N. & Helleday T. Hydroxyurea-stalled replication forks become progressively inactivated and require two different RAD51-mediated pathways for restart and repair. Mol Cell 37, 492–502 (2010). 20188668 10.1016/j.molcel.2010.01.021 PMC2958316

[b56] Wang Y. & Qin J. MSH2 and ATR form a signaling module and regulate two branches of the damage response to DNA methylation. Proc Natl Acad Sci USA 100, 15387–15392 (2003). 14657349 10.1073/pnas.2536810100 PMC307577

[b57] Sollier J. . Transcription-coupled nucleotide excision repair factors promote R-loop-induced genome instability. Mol Cell 56, 777–785 (2014). 25435140 10.1016/j.molcel.2014.10.020 PMC4272638

[b58] Dominguez-Sanchez M. S., Barroso S., Gomez-Gonzalez B., Luna R. & Aguilera A. Genome instability and transcription elongation impairment in human cells depleted of THO/TREX. PLoS Genet 7, e1002386 (2011). 22144908 10.1371/journal.pgen.1002386 PMC3228816

[b59] Bianco J. N. . Analysis of DNA replication profiles in budding yeast and mammalian cells using DNA combing. Methods 57, 149–157 (2012). 22579803 10.1016/j.ymeth.2012.04.007

[b60] Bayani J. & Squire J. A. Sister chromatid exchange. Curr Protoc Cell Biol Chapter 22, Unit 22, 27 (2005). 10.1002/0471143030.cb2207s25 18228468

