# Supplementary material for: Roles of human POLD1 and POLD3 in genome stability
Source: Sci Rep. 2016 Dec 15;6:38873. doi: 10.1038/srep38873 (PMC5156928; doi:10.1038/srep38873)
Supplement: Supplementary Information [file srep38873-s1.pdf]

SUPPLEMENTARY INFORMATION

Roles of human POLD1 and POLD3 in genome stability

Emanuela Tumini, Sonia Barroso, Carmen Pérez Calero and Andrés Aguilera

SUPPLEMENTARY METHODS

Cell proliferation

After siRNA transfection, HeLa cells were seeded in 96 well-plate at a density of 2000 cells per well in triplicate and cell proliferation was measured at different time-points by WST-1 reagent (Roche) following manufacturer's protocol. 4 hours after WST-1 addition, absorbance was read using a Varioskan Flash Multimode Reader (Thermo Scientific).

Antibodies

For Western blot analysis, anti-HA-tag antibodies (11867423001) were purchased from Abnova. For immunofluorescence, antibodies anti-POLD3 (ab182564) were purchased from Abcam.

HR assay

MCF7-DRGFP cells and retroviral plasmid to produce I-*Sce*I (pMXPIE-ISceI) were a kind gift from Fernandez-Capetillo's laboratory [1]. Retroviruses were produced in HEK 293T cells using standard procedures. MCF7-DRGFP cells were siRNA transfected as described above and, at 72 hours from the first transfection, they were transduced using retroviral supernatant. 72 hours after transduction, the percentage of GFP expressing cells was assessed by flow cytometry (FACSCalibur; BD).

Clonogenic assay

After siRNA transfection, HeLa cells were seeded in 6-cm dishes at low density, 400 cells each dish. Cells were treated with increasing doses of HU or CPT or left untreated; each condition was plated in triplicate. After 24 hours of treatment, CPT containing medium was removed, cells were washed once with PBS and released in fresh medium. Cells were grown for approximately 2 weeks to allow the formation of single cell colonies. Colonies were subsequently fixed with methanol, stained with 1% crystal violet, rinsed with tap water and counted. The number of colonies for each siRNA-mediated depletion was normalized with its untreated control.

siPOLD3 rescue experiments

Of the four siRNAs constituting the targeting pool, one siRNA annealing to the 3' UTR of the *POLD3* gene was purchased separately from MWG. HeLa cells were transfected as already described in the "siRNA transfection" section to knock down the levels of POLD3. Following 24 hours from the second reverse transfection cells were transfected with a plasmid containing only the POLD3 coding sequence (GeneCopoeia, Cat.no EX-Y2063-M02), being therefore resistant to the 3' UTR siRNA-dependent gene silencing. At 24 hours after plasmid transfection, cells were fixed by 4% formaldehyde and standard immunofluorescence was perform to stain γH2AX and POLD3.

Real-time qPCR

cDNA was synthesized by reverse transcription using QuantiTect Reverse Transcription Kit (Qiagen). RT-qPCR was performed with iTAq Universal SYBR Green Supermix (Bio-Rad) using 7500 Fast-Real-Time PCR System. The analysis

was performed with 7500 software v2.3. Primers used were REV3L Fwd (5'-TGC CCC AAG TCG ACA ACT G-3'), REV3L Rev (5'-TTG GAA CGT TCG TAT TCT TCT TTG-3'), POLD3 Fwd (5'-GCC TCT GTT CAA TAC TGA CTA TGA CAT C-3') and POLD3 Rev (5'-GAC GGG AGC TGC ACA TTG TA-3').

SUPPLEMENTARY REFERENCE

1. Murga, M. *et al.* Global chromatin compaction limits the strength of the DNA damage response. *J Cell Biol.* **178**, 1101-1108, (2007).

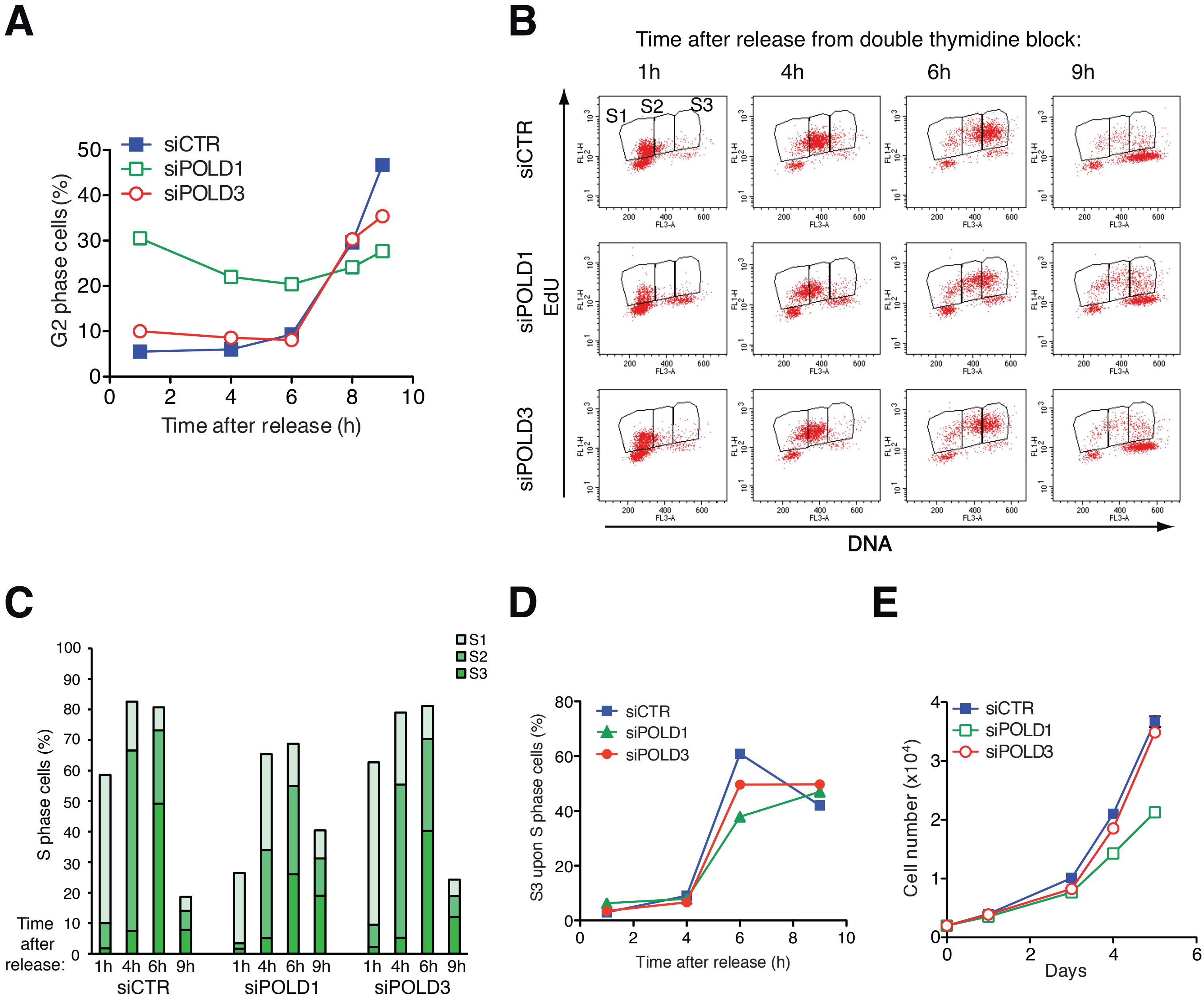

Supplementary Figure S1. Cell cycle progression and cell proliferation of POLD1- and POLD3-depleted cells. **(A)** Percentage of cells that progress into G2 phases based on Fig. 1C. **(B)** FACS profile of HeLa cells at different time points after release from double thymidine block. S-phase cells were gated in three different regions corresponding to early (S1), middle (S2) and late (S3) S-phase accordingly with the DNA content. **(C)** Percentage of cells that progress into S1, S2 or S3 regions based on panel B. **(D)** Percentage of S-phase cells that progress into S3 based on panel B. **(E)** HeLa cell growth curves after siRNA transfection.

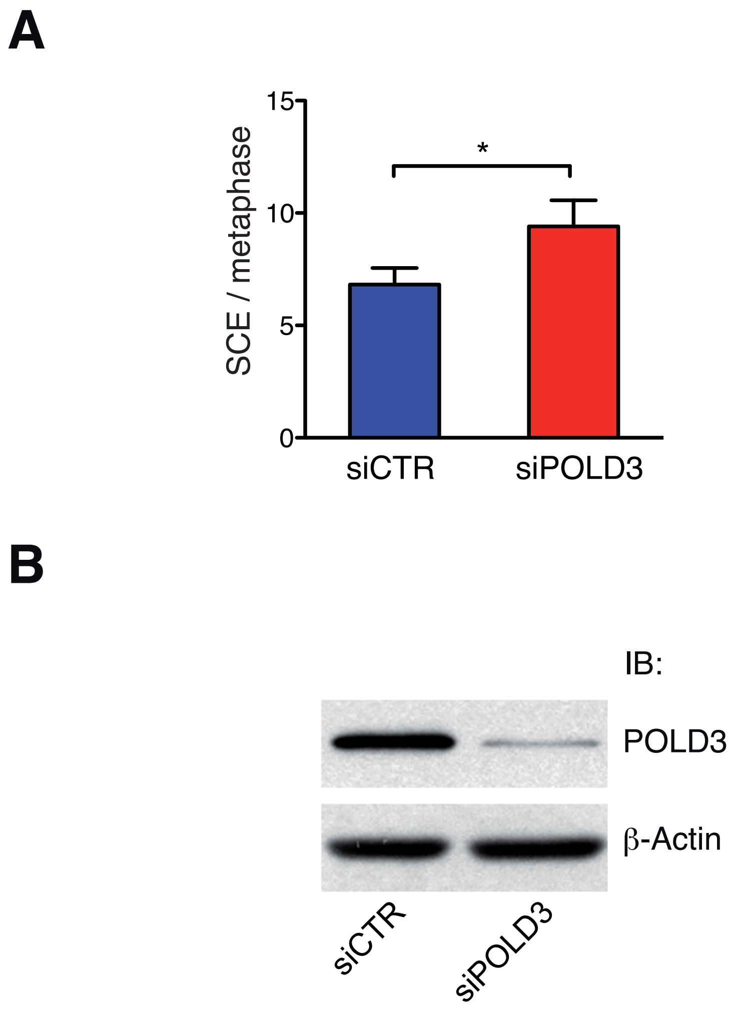

Supplementary Figure S2. Role of pol δ on SCE. (A) Frequency of SCE per metaphase in U2OS after POLD3 depletion. At least 10 metaphases were scored for each experiment. Means ± SEM from five independent experiments are shown. Differences between distributions were assessed by paired t-tests. **(B)** Immunoblot to assay the knockdown of the siRNA targeted protein. β-Actin was used as a loading control.

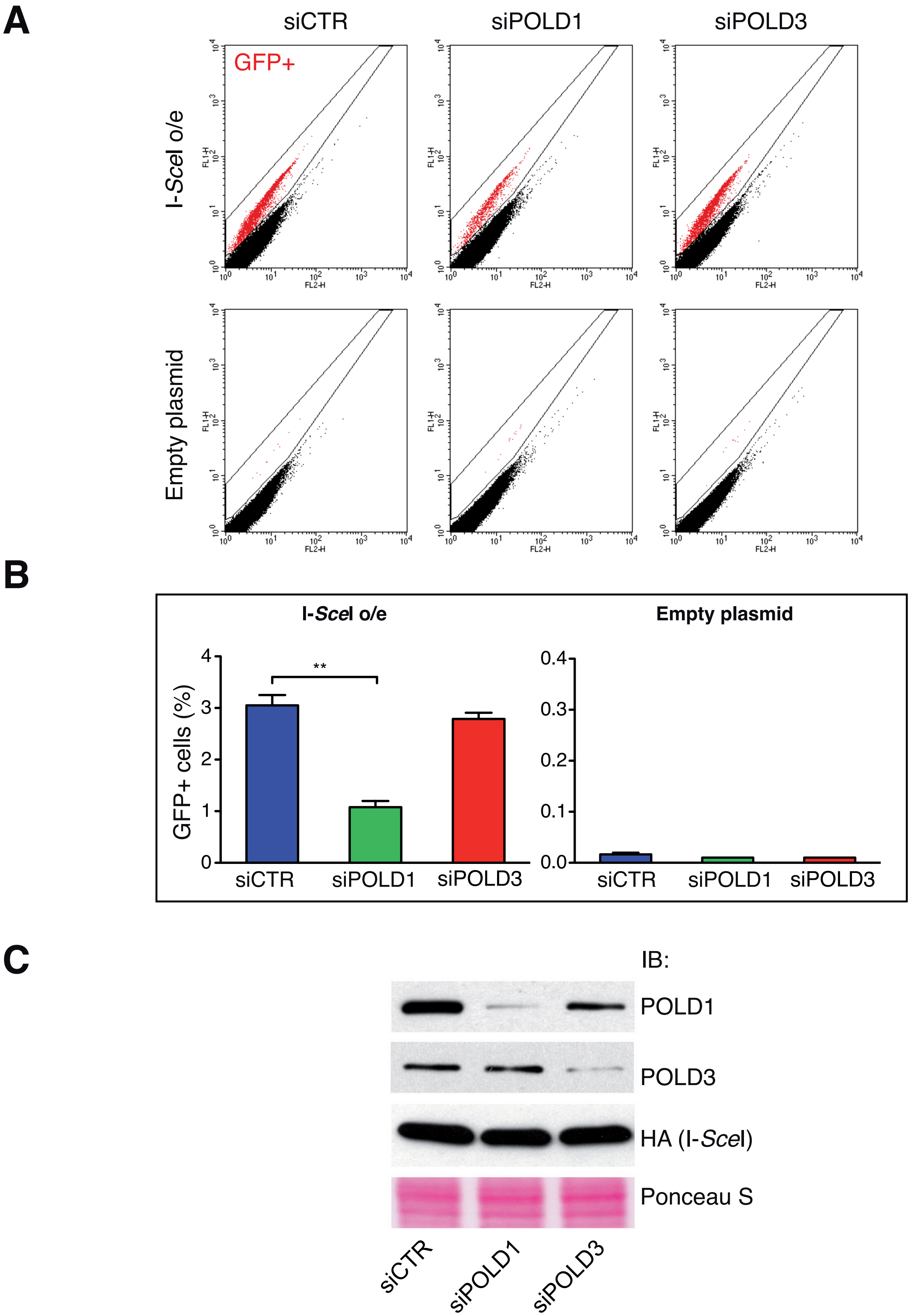

Supplementary Figure S3. Role of pol δ on HR repair. (A) FACS profile from one representative experiment. MCF-7 DR-GFP cells were transfected to overexpress the I-*Sce*I endonuclease or with an empty plasmid and the frequency of restored GFP signal was assessed by flow cytometry. **(B)** Percentage of GFP positive cells. Means ± SEM from three independent experiments are shown. Differences between distributions were assessed by paired t-tests. **(C)** Immunoblot to assay the knockdown of the siRNA targeted protein. Overexpression of the HA-tagged I-*Sce*I endonuclease was also verified. Ponceau-S staining was used as a loading control.

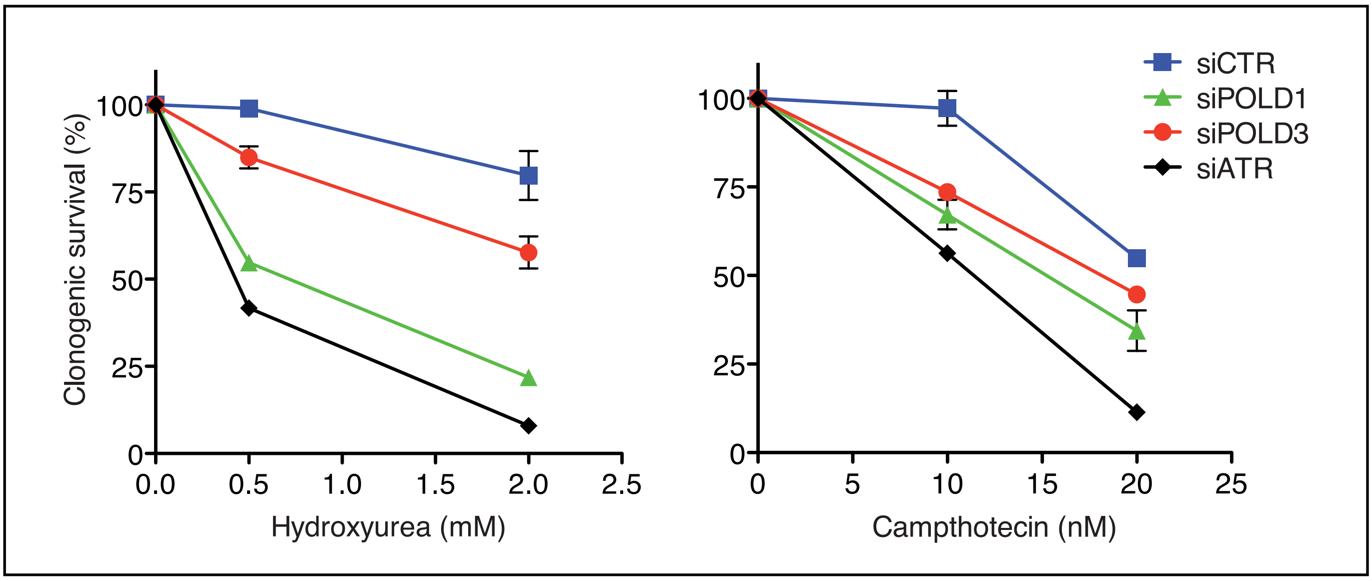

Supplementary Figure S4. Increased sensitivity to HU or CPT assessed by clonogenic assay. After siRNA transfection, HeLa cells were seeded at low density, treated with increasing doses of HU or CPT and grown for approximately 2 weeks to allow the formation of single cell colonies. Colonies were subsequently fixed, stained and counted.

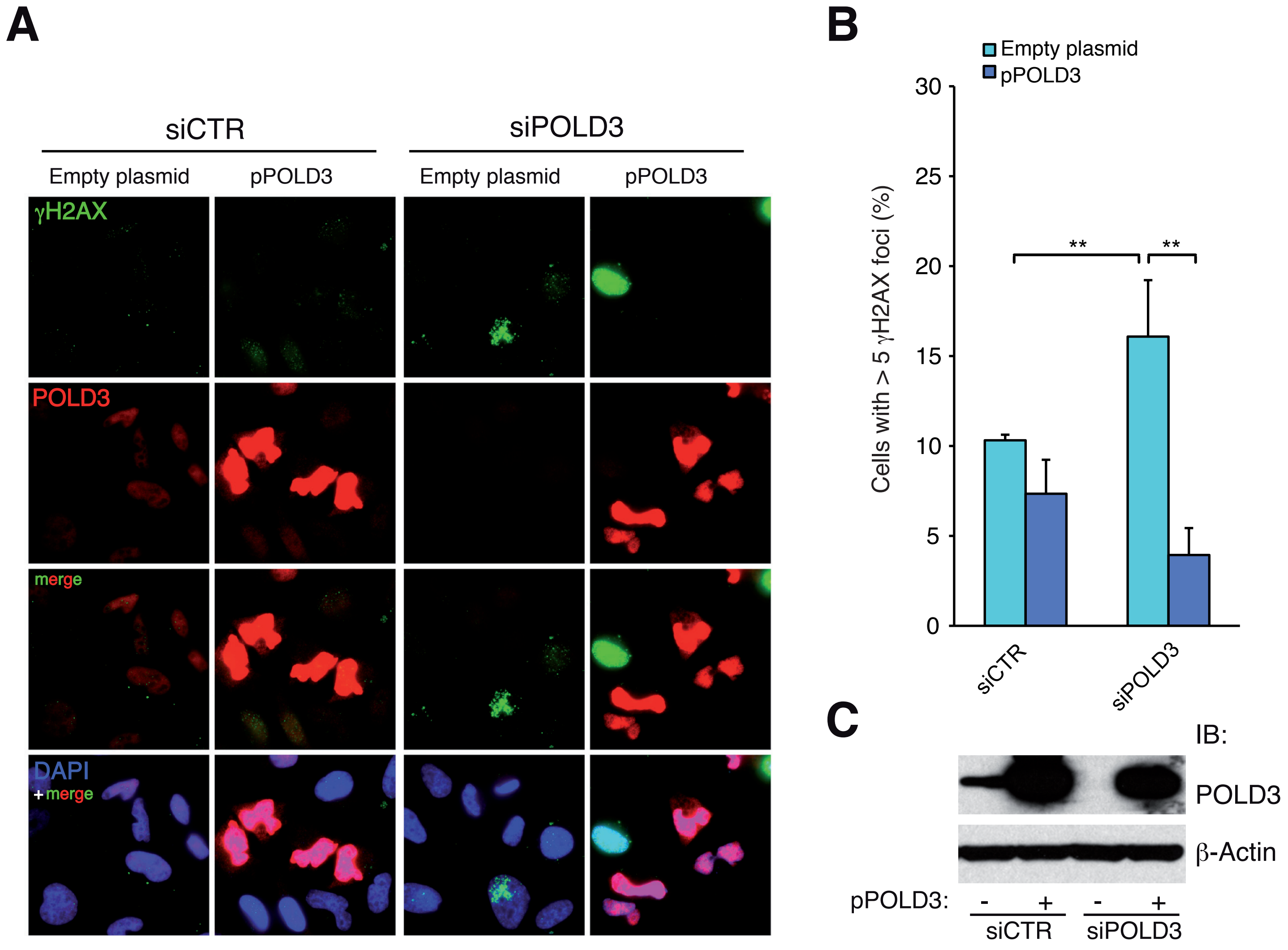

Supplementary Figure S5. Ectopic expression of POLD3 rescued the siPOLD3-dependent γH2AX foci accumulation. (A) Immunofluorescence of HeLa cells stained with antibodies against γH2AX and POLD3. HeLa cells were transfected with the empty plasmid or the pPOLD3 plasmid (EX-Y2063-M02) for POLD3 siRNA-resistant expression. **(B)** Percentage of cells with more than 5 foci. More than 80 cells expressing POLD3 (positive-stained) or more than 100 cells of mixed population transfected with the empty plasmid were counted in each experiment. Means ± SEM from three independent experiments are shown. Differences between distributions were assessed by the Mann-Whitney test. **(C)** Immunoblot to assay the knockdown and the ectopic expression of POLD3. β-Actin was used as a loading control.

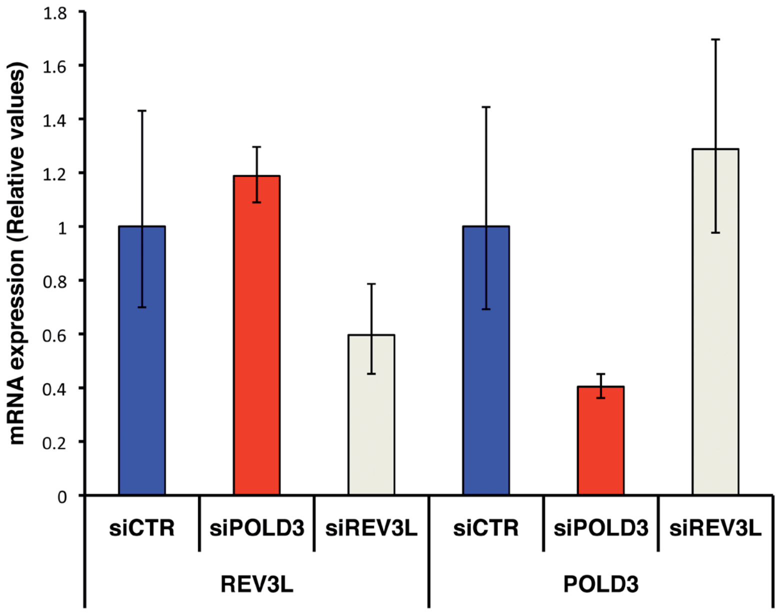

Supplementary Figure S6. REV3L and POLD3 mRNA expression levels. The graph shows the relative mRNA expression levels in siREV3L and siPOLD3 HeLa cells. Error bars show the minimum and maximum RQ values.
